# Toward explainable heat load patterns prediction for district heating

**DOI:** 10.1038/s41598-023-34146-3

**Published:** 2023-05-08

**Authors:** L. Minh Dang, Jihye Shin, Yanfen Li, Lilia Tightiz, Tan N. Nguyen, Hyoung-Kyu Song, Hyeonjoon Moon

**Affiliations:** 1 Department of Information and Communication Engineering and Convergence Engineering for Intelligent Drone, Sejong University, Seoul, Republic of Korea; 2 Department of Artificial Intelligence, Sejong University, Seoul, Republic of Korea; 3 Department of Computer Science and Engineering, Sejong University, Seoul, Republic of Korea; 4 School of Computing, Gachon University, 1342 Seongnam-daero, Sujeong-gu, Seongnam-si, Gyeonggi-do 13120 Republic of Korea; 5 Department of Architectural Engineering, Sejong University, 209 Neungdong-ro, Gwangjin-gu, Seoul, 05006 Republic of Korea

**Keywords:** Energy harvesting, Mathematics and computing

## Abstract

Heat networks play a vital role in the energy sector by offering thermal energy to residents in certain countries. Effective management and optimization of heat networks require a deep understanding of users' heat usage patterns. Irregular patterns, such as peak usage periods, can exceed the design capacities of the system. However, previous work has mostly neglected the analysis of heat usage profiles or performed on a small scale. To close the gap, this study proposes a data-driven approach to analyze and predict heat load in a district heating network. The study uses data from over eight heating seasons of a cogeneration DH plant in Cheongju, Korea, to build analysis and forecast models using supervised machine learning (ML) algorithms, including support vector regression (SVR), boosting algorithms, and multilayer perceptron (MLP). The models take weather data, holiday information, and historical hourly heat load as input variables. The performance of these algorithms is compared using different training sample sizes of the dataset. The results show that boosting algorithms, particularly XGBoost, are more suitable ML algorithms with lower prediction errors than SVR and MLP. Finally, different explainable artificial intelligence approaches are applied to provide an in-depth interpretation of the trained model and the importance of input variables.

## Introduction

District heating (DH) has risen as a crucial energy supply infrastructure in order to effectively provide heat and cooling to consumers over the last few decades^1^. DH is superior in many aspects compared to other energy supply options, which include having a lower carbon footprint, the integration of multiple heat sources, and high energy throughput. The latest fourth and fifth generations of DH can utilize several heat sources, which include combined heat and power (CHP), gas boilers, water-source heat pumps (HPs), ground-source HPs, and solar energy-based HPs. The recent literature focused more on developing simulation frameworks and effective approaches in regards to designing and optimizing DH systems in terms of the economic and energetic factors, which is due to the fast development of DH technologies^2,3^. Storage technology is also a hot topic, because it helps decouple heat production and the demand to increase DH efficiency^4^. The following articles^1,5^ were reviewed in order to obtain the latest information about DH networks.

The heat usage pattern analysis has become increasingly essential as the number of end-users increases, because it greatly impacts the entire network's efficiency. Variations in the heat usage behavior from the consumers' side lead to variations in the heat usage pattern of a single substation, which is a major matter for accurate and efficient DH management and operation^6^. For example, the substantial temperature difference between the summer and the winter significantly influences the users' heat demand. In addition, the hourly heat demand also varies between households, which causes heat demand variation at the substation^7^.

An accurate heat demand prediction framework is imperative in order to effectively manage DH networks^8^. First, it facilitates the optimization of the overall heat production, minimizes the heat loss, and optimizes the operating costs. Second, the distribution temperature is provided at an appropriate range in order to predict the real-time heat usage using the heat demand forecast model. As a result, the number of studies proposed in regards to predicting the heat demand has been increasing. A heat demand analysis can generally be divided into model-based and data correlation categories^9^. The data correlation approach mainly depends on building functional correlations of the DH parameters in order to develop a heat usage profile for each substation or building. The model-based technique relies on machine learning (ML) algorithms in order to effectively learn the representative patterns using the historical heat load data^10^. The data correlation approach offers higher accuracy than the model-based approach, but it is time-consuming and laborious due to each building/substation having a unique heat usage profile that needs to be constructed. The performance of the model-based heat usage prediction algorithm has become significantly better, which is due to the huge advancements in artificial intelligence (AI) and big data over the past few decades^9,10^.

The heat usage prediction, heat loss estimation, and abnormality analysis based on the energy signature (ES) have been increasingly investigated in recent years, which have shown promising results^11,12^. However, these studies mainly used outdoor temperature as the main feature in order to discover the heat demand pattern. Other studies focused on peak usage forecasting with the ultimate objective of optimizing the energy usage and DH management^13^. These studies, which are similar to the ES, failed to consider the meteorological data or the end-user behaviors. Potential influencers of the heat demand patterns can be divided into three main factors, which include meteorology, behaviors, and time^14^. Some common meteorological data that potentially affects heat demand are humidity, solar irradiation, outdoor temperatures, and the wind flow speed^15^. Time factor involves all time-related parameters, which include hours, days, months, and years. The social behaviors of the end-users are also a crucial influencer of the heat load variation, which can be affected by both meteorological and time factors^16^. These three main factors significantly influence the heat demand patterns.

There has been considerable interest in the research area of heat load forecasting for DH, as indicated by numerous recent studies. Idowu et al.^17^ examined a range of supervised ML algorithms in order to perform heat load prediction up to 48 h in advance. The experimental results revealed that conventional ML algorithms, such as SVM and linear regression, achieved the lowest normalized root mean square error when compared to other algorithms. In another study, Boudreau et al. found that ensemble models provided significantly better prediction accuracy than base ML models when it came to predicting peak power demand and next-day building energy usage^18^.

Several studies have delved into specific aspects of DH systems. For example, Saloux et al. explored the application of ML algorithms for predicting the aggregated heating usage of a community. They concluded that the models' performance could be significantly enhanced by considering other crucial factors, such as time of day, systematic variables, and temperature^19^. López et al. focused on the impact of specific days, such as holidays or festive periods, on the load curve, and determined that such events could considerably affect the heat usage pattern^20^. Moreover, a case study of a large DH network over several heating seasons revealed that the primary force of heat demand were the various operation settings during daytime (night shutdown and night temperature setback) and the outdoor temperature^21^.

Despite the numerous issues addressed and methods discussed in existing literature on heat load prediction in DH networks, further research is needed to explore important external factors such as holiday and weather conditions, which could be utilized as input to improve the models' accuracy^6^. Additionally, while previous work has showed the high predictive performance of ML algorithms for heat demand, they have not provided a clear explanation of why the model achieved good performance, as well as which features are important and their correlation with the models^10^.

This research is proposed in order to improve the heat usage prediction via an in-depth analysis of the dataset to figure out the potential factors that impact the heat demand. The main contributions include (a) performing a data analysis prior to the training process to help thoroughly understand the dataset, (b) training and comparing different ML models in order to obtain the best hourly heat load prediction model, and (c) offering detailed explanations about what features were imperative to the model prediction, which were overlooked in the previous studies.

The remainder of the manuscript is outlined as follows. Section “Dataset description” gives a detailed description of the proposed heat demand dataset. After that, the Section “Methodology” outlines all processes involved in heat demand prediction. Several experiments are performed in Section “Experimental results” to comprehensively assess the proposed framework. Next, the Section “Discussion” discusses the findings and provides a detailed analysis of the study. Finally, we conclude the study and offer future work in the Section “Conclusion”.

### Dataset description

The dataset that is described in this research was the hourly heat demand from an eco-friendly liquefied natural gas (LNG)-based cogeneration plant in the Cheongju region, Korea. The plant produces around 76.5 Gigacalories (Gcal) of local heating to the distribution grid. Gcal is a common heat load unit, which measures the heat energy in the heating plants. The LNG-powered plant is more efficient and environmentally friendly for the generation of thermal energy, which has been reported to produce over 70% less emission than coal or oil sources.

The dataset introduced in this study includes the hourly heat usage from January 2012 to December 2020 of the residents from a region, which spans eight heating seasons from November to April. The heat usage profile suggests the amount of heat that is transmitted from the plant to the consumers at a specific duration, which mainly involves space heating (SH) and domestic hot water (DHW). The corresponding hourly historical weather data was also collected as an additional feature in order to discover the potential connections with the heat load patterns in addition to the heat load data. A holiday feature that indicates whether the day under consideration is a holiday is also added in order to investigate the end-user behaviors. The three main features that belong to the weather data include wind flow speed, humidity, and outdoor temperature. The collected heat usage dataset is used to study the hourly heat load patterns and provides some explanations for the model's predictions. The minimum, maximum, mean and standard deviation for each variable are described in Table 1.

**Table 1:** Description of important observations with possible values for the variables in the proposed dataset.

Name	Minimum|maximum	Mean|standard deviation	Unit
Date	01/01/2012|01/01/2022	–	–
Wind speed	0|8.7	1.47|0.93	m/s
Humidity	7|100	61.32|20.02	%
Outdoor temperature	− 16.5|38.1	13.75|10.83	°C
Holiday	0 (normal day)|1 (holiday)	0.32|0.46	–
Heat load	0|317	65.89|52.92	Gcal

In summary, 8760 hourly heat load profiles and their corresponding historical temperature data are obtained yearly. Therefore, a total of 87,672 entries, which include date and time, holiday, wind flow speed, humidity, and temperature, are used as the input variables, and the heat load profiles are used as the target variables. The data entries from 2012 to 2020 were used as the training set, whereas the hourly heat usage of 2021 was applied in order to test the model’s performance.

## Methodology

**Figure 1:**
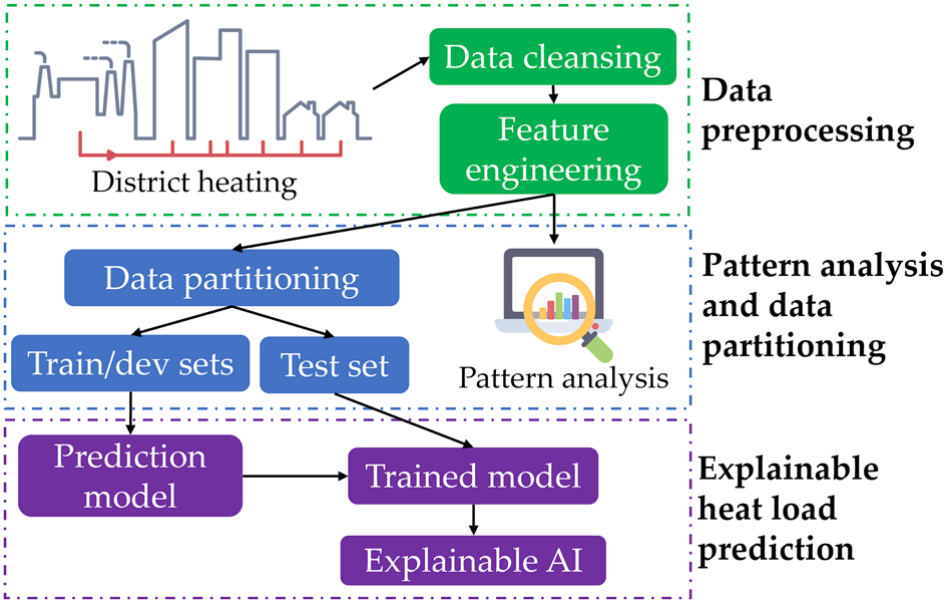
Description of the primary components of the heat usage patterns analysis framework.

Figure 1 depicts the three components of the hourly heat usage prediction system, which are (a) data preprocessing, (b) pattern analysis and data partitioning, and (c) explainable heat load forecasting.

- Data preprocessing: There is a high possibility that the structured data may contain some common issues with data preprocessing, such as duplicate data, missing data, and negative data due to human errors, which can affect the system's performance. As a result, it is a prerequisite before the data analysis and training processes to fix all errors and standardize the data.
- Pattern analysis and data partitioning: Heat usage patterns play an important role in regards to enabling specialists to study consumer behavior. The distinctive patterns of the dataset are discovered in this section by using various data analysis approaches in order to thoroughly analyze the dataset before the training phase. The dataset is then divided into training and testing sets.
- Explainable heat load prediction: Different ML algorithms were trained in order to forecast the hourly heat usage. Some explainable artificial intelligence (XAI) approaches are finally implemented in order to interpret the model’s predictions.

### Data preprocessing

#### Data cleaning

The structured data-related issues, such as missing and duplicated data are unavoidable during the data collection, and they can negatively affect the model's performance if not appropriately corrected. Data cleansing is therefore conducted in order to detect and fix error records in regards to the humidity, wind speed, outdoor temperature, and hourly heat usage data. There are various data cleaning processes, and the two main processes that were performed in this study include removing duplications and fixing the missing values. The dataset is loaded as a data frame using pandas, a famous data manipulation and analysis library. After that, data inconsistencies can be automatically detected using pandas-supported functions.

Standard techniques, such as moving average (MA) and imputation, are usually employed in order to correct the missing data. This study applied the exponential weighted moving average (EWMA) technique^22^, which is an extension of the MA algorithm. EWMA considers the recent data points to be significantly important with a higher weight, whereas the data points in the further past receive an exponentially lower weight. Moreover, the EWMA method can be effectively applied due to the nature of the dataset, and the differences between the two consecutive data points are considered minor. The EWMA can be described as follows.

1 $${E}_{t}=\alpha \times {x}_{t}+(1-\alpha )\times {E}_{t-1}$$

where ${E}_{t}$ indicate the computed value at time t based on the EWMA technique. ${x}_{t}$ is the value of the series in the current period. ${E}_{t-1}$ is the EWMA at the previous time period. Finally, $\alpha$ is the smoothing factor, which ranges between 0 and 1 and controls the influence of the current value ${x}_{t}$ on the ${E}_{t}$. A larger $\alpha$ places more weight on recent observations and results in a more reactive EWMA, while a smaller $\alpha$ results in a smoother EWMA.

#### Feature engineering

Feature engineering is the process of selecting, extracting, and transforming relevant features or variables from raw data to enhance the performance of ML algorithms. The goal of feature engineering is to provide ML algorithms with informative and discriminative features that can help them better understand the underlying patterns and relationships in the data. Two main processes in the feature engineering process are standardization and feature transformation.

The regression model fitting and learned function can be negatively affected by structured data, and it eventually creates a bias when numerical features with different scales are fed into the model^23^. The normalization/standardization techniques therefore need to be implemented in order to normalize the input features. Min–max normalization and standardization are two common feature scaling approaches^24^. The heat usage dataset that is applied to fit the model contains peak heat load on some specific periods, which are outliers, and it has an essential role during the training process. The min–max normalization likely lowers the impact of those outliners by transforming all features into a range between 0 and 1. The standardization therefore scales the features in order to have a zero mean, and a standard deviation of 1 is implemented in this study.

Feature transformation is necessary for structured data in order to convert categorical inputs into numerical inputs, because most ML models work with numerical data. The holiday variable is categorical, because it has two distinctive values, which represent whether a particular day is a regular day or a holiday. As a result, one-hot encoding, which creates a binary representation of the categorical feature, is applied in order to transform the holiday feature^25^. For instance, when a specific day is a holiday, the value for the holiday binary variable is set to 1, and the regular binary variable is 0.

### Pattern analysis and data partitioning

#### Pattern analysis

##### Heat network during the summer season

**Figure 2:**
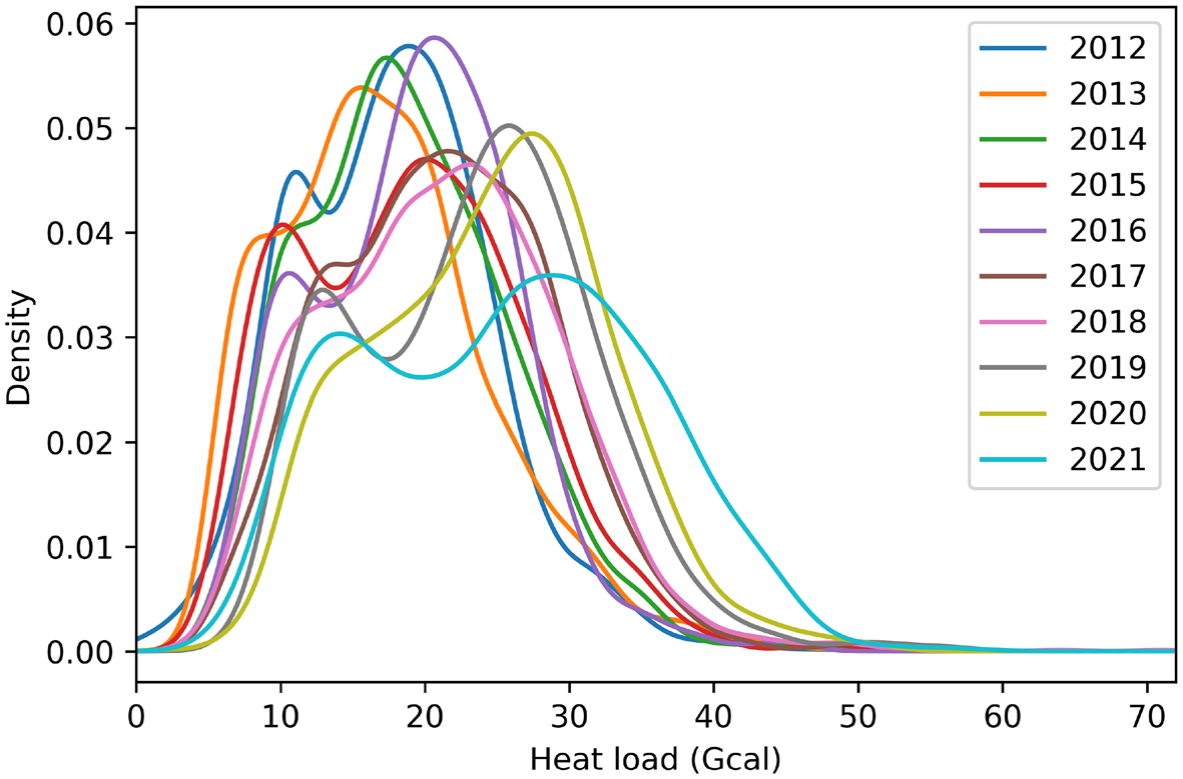
Distribution density plot of hourly heat demand during the summer season (Jun.–Aug.).

The investigation of the heat network in the summer season, which spans from June to August, gives some exciting insights into the town's heat usage. Figure 2 illustrates the hourly heat demand distribution density for the summer months from 2012 to 2021. The average heat demand in the summer mainly involves the DHW consumption and the network heat losses. It can generally be seen that there was less heat demand in the distant past compared to the recent years. For instance, a roughly similar distribution can be observed for the following years, which include from 2012 to 2016, with the average heat demand being around 20 Gcal. However, the average heat demand increased to around 30 Gcal, which included the more recent years from 2019 to 2021, with some higher heat demands being related to particular heat usage patterns. Moreover, there has been a gradually increasing trend in the average heat usage of over 40 Gcal in recent years, and the year 2021 shows the highest density.

##### Heat network during the winter season

**Figure 3:**
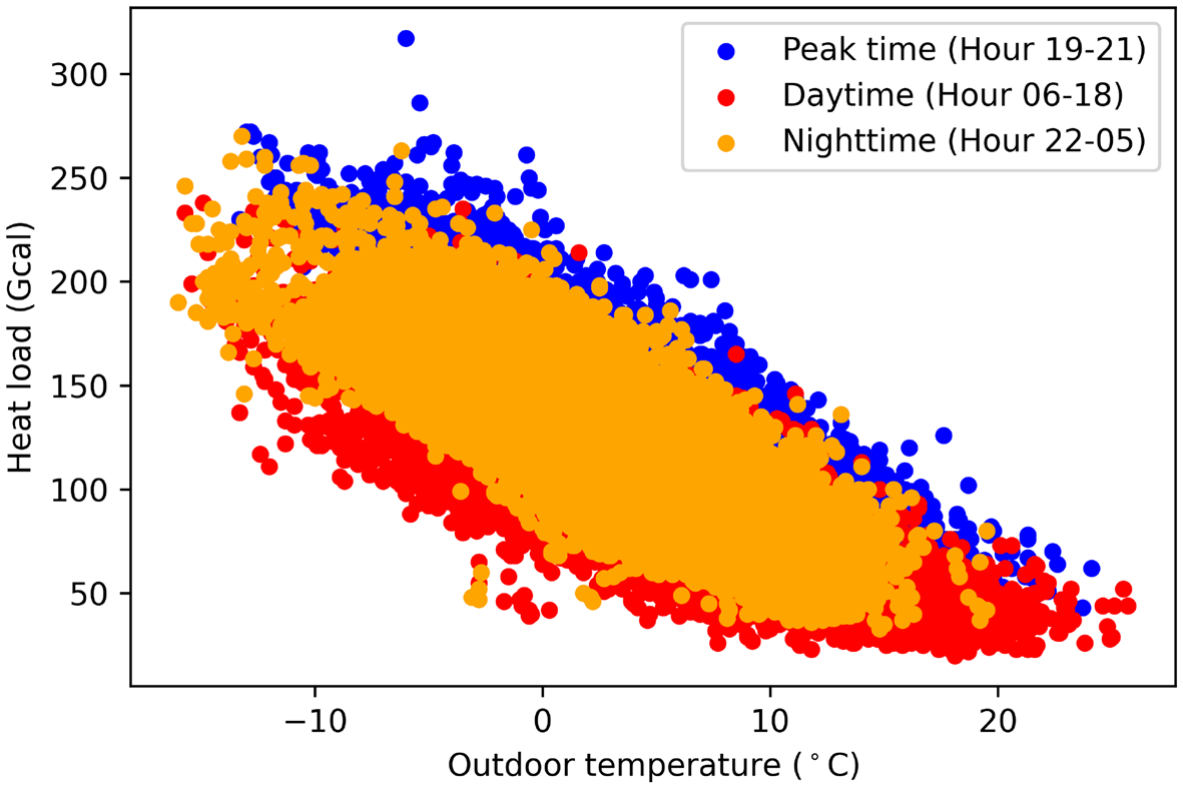
Scatter plot of the outdoor temperature and the heat usage during the winter season (Nov.–Mar.).

The chart in Fig. 3 illustrates the network's energy consumption on an hourly basis during the winter season spanning from November to March. The chart depicts three distinct patterns for three different time periods: daytime (06:00–18:00), nighttime (22:00–05:00), and peak hours (19:00–21:00). The scatter plot reveals that the consumers tend to use more heat during the peak time at the same temperature level compared to the nighttime and daytime. Moreover, the lower the outside temperature, the higher the heat load that is required.

##### Some heat load patterns for each season of the year

**Figure 4:**
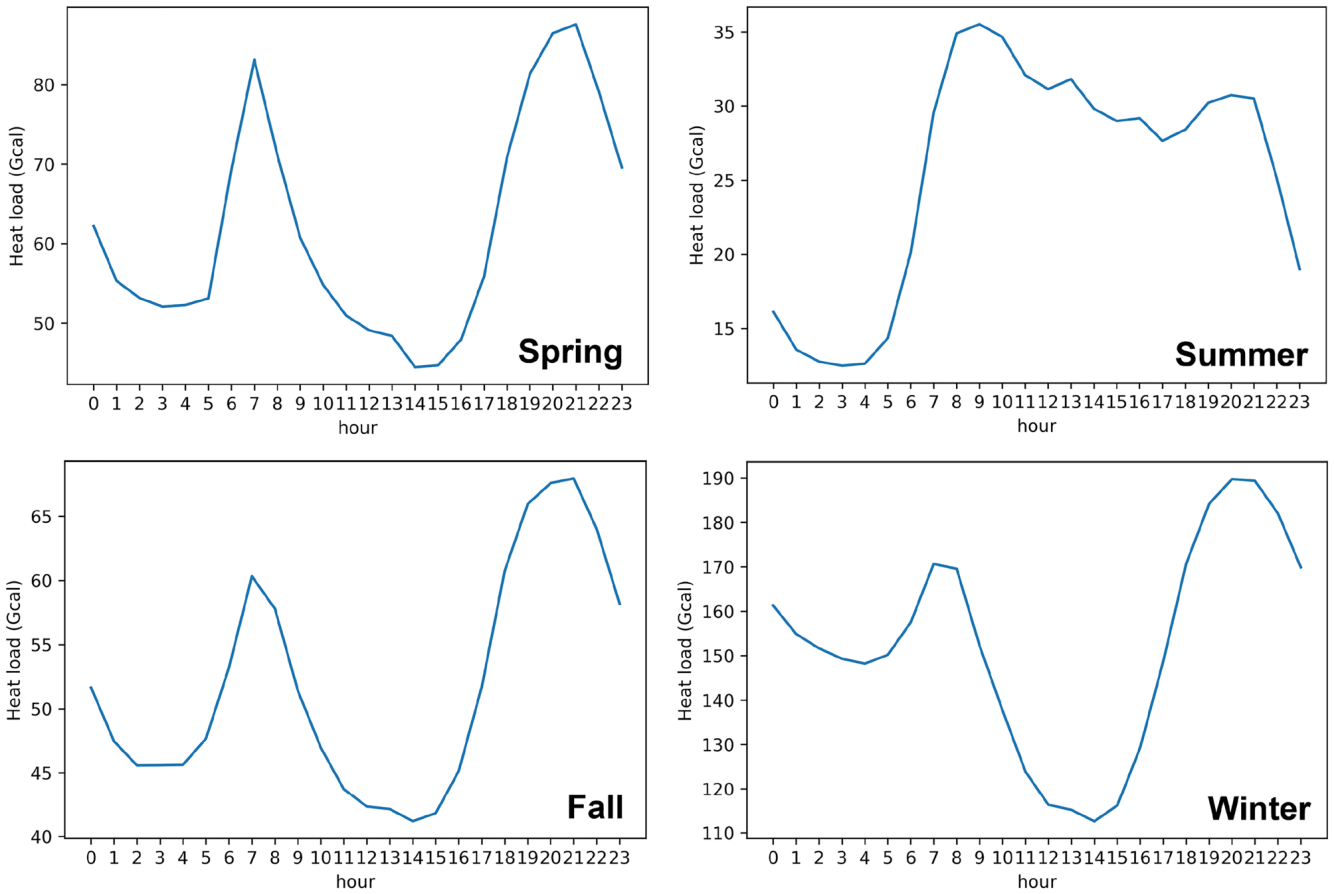
Average weekly heat load patterns during the four season periods.

A typical hourly heat load pattern for each season can be observed in Fig. 4. The spring, fall, and winter seasons have similar variations in the hourly time scale, which is caused by the social behavior of the end-users. Reduced heat loads can be observed in the daytime, which is due to solar radiation that leads to higher daytime temperatures. The highest heat load during the daytime occurs around 8 am in order to prepare the space heating in offices and commercial buildings. The heat demand usually peaks between 19:00 and 21:00 because of the low temperature at night, which requires more heat for SH and DHW. DHW is a major part of the heat demand in the summer, when a tiny difference in the heat variation can be observed.

#### Data partitioning

Data partitioning is a fundamental step required before training and evaluating the model. After preprocessing, the data is split into two sets: the training set and the testing set. The training set is utilized to train and optimize the model, while the testing set is typically employed to assess the algorithms' performance across various scenarios. This study used the heat usage profiles between 2012 and 2020 as the training set, whereas the heat load profiles from 2021 were used for the testing. Each training or testing sample consists of day, hour, outdoor temperature, humidity, windspeed, and holiday as the input variables, while the output is the hourly heat usage corresponding to that particular input.

### Explainable heat load prediction

This section presents the main concepts behind boosting, support vector regression (SVR)^26^, and multilayer perceptron (MLP) algorithms^27^ that were implemented for the heat demand forecasting.

#### Boosting algorithms

Boosting algorithm belongs to the ensemble approach, which sequentially adds multiple weak learners. Each weak learner is added by using the learned information from its predecessor, and it tries to correct the errors that are predicted by them. A weak learner can be any learning algorithm that offers a slightly better performance than random guessing. Two standard boosting approaches are gradient boosting and adaptive boosting^28^.

- Adaptive boosting: The adaptive boosting (AdaBoost) algorithm was proposed by sequentially adding weak learners, which involved using decision trees, and attempting in order to correct the wrongly predicted samples by applying a bigger weight to them during the training process of the latter weak learners. The AdaBoost model's final output is the weighted median.
- Gradient boosting: AdaBoost assigns new instance weights whenever a new weak learner is added, but gradient boosting aims to fit the new predictor to the residual errors that are caused by the prior predictor with the primary objective of minimizing a loss function^29^. Some popular gradient boosting algorithms include LightGBM and XGBoost.

XGBoost leverages the feature distribution across all data points to narrow down the search space of potential feature splits. The objective of the XGBoost algorithm can be expressed as:

2 $$objective=L+\mu $$

where the predictive ability of XGBoost is determined by the loss function $L$, while the regularization term $\mu$ is used to manage overfitting. $\mu$ is determined by the number of observers and their prediction threshold in the ensemble model. Since the problem in question belongs to regression analysis, the root mean squared error (RMSE) is used as the loss function $L$.

#### Support vector regression (SVR)

Unlike typical regression algorithms that seek to minimize the sum of squared errors between actual and predicted values, SVR attempts to identify the optimal hyperplane within a user-defined threshold value. The threshold value is the distance between the boundary line and the hyperplane. Heat demand prediction is a complex non-linear topic, because it has multiple input variables. To address non-linearity in the initial feature space and treat it as a linear problem in the high-dimensional feature space, SVR requires the use of a non-linear kernel. The Gaussian Radial Basis kernel (RBF) was used in this study as the default kernel for SVR.

#### Multilayer perceptron (MLP)

Multilayer perceptron (MLP) belongs to the feedforward artificial neural networks (ANN) category. MLP's fundamental structure consists of an input layer, one or more hidden layers with neurons, and an output layer that are stacked in sequence. The neuron is the primary computing component of MLP, and neurons from the current layers fully connect to neurons from the next layer. The inputs are added to the initial weights, fed into an activation function, and propagated to the next layer.

## Experimental results

This section shows all experiments that were conducted to determine the most suitable algorithm for predicting heat usage. In addition, various XAI techniques were also conducted in order to provide an in-depth analysis of the trained models.

The heat load prediction models were constructed and trained on scikit-learn^30^, a Python-based open-source ML library. Three main explainable AI libraries for analyzing the data include partial dependence plot^31^ (PDP), which is a global and model-agnostic XAI algorithm, local interpretable model-agnostic explanations^32^ (LIME), which create a local model approximation of the model around the prediction of interest, and shapley additive explanations^33^ (SHAP), which employ a game-theoretic approach.

### Evaluation metrics

Three standard evaluation metrics were computed, which included the coefficient of determination (${R}^{2}$), mean squared error (MSE), and mean absolute error (MAE) in order to evaluate the heat demand forecasting. MSE is computed by averaging the squared difference between the predicted values and actual values for all the training samples^34^. On the other hand, MAE is the average of the absolute differences between the predicted values and true values. While MSE measures the standard deviation of residuals, MAE calculates the average of the residuals in the dataset. ${R}^{2}$ is computed by determining the proportion of the dependent variable's variance predicted by the algorithm. The lower the MSE and MAE scores, the better the model’s performance. However, a higher value of ${R}^{2}$ is considered better. The three metrics can be formulated as follows.

3 $$MSE=\frac{1}{N}{\sum }_{i=1}^{N}{\left({y}_{i}-{\widehat{y}}_{i}\right)}^{2}$$

4 $${R}^{2}= 1-\frac{\sum {\left({y}_{i}-{\widehat{y}}_{i}\right)}^{2}}{\sum {\left({y}_{i}-\overline{y }\right)}^{2}}$$

5 $$MAE=\frac{1}{N}{\sum }_{i=1}^{N}\left|{y}_{i}-{\widehat{y}}_{i}\right|$$

where $N$ is the total number of training samples. ${y}_{i}$ indicates the actual value, ${\widehat{y}}_{i}$ means the predicted value of the $i$ th profile, and $\overline{y }$ is the mean value of $y$.

### Hyperparameter fine-tuning

Five regression models were implemented in this study in order to perform the heat demand forecasting, which included SVR, AdaBoost, XGBoost, LightGBM, and MLP. Each model has its crucial hyperparameters that must be determined before the training. The hyperparameters control the training behavior of the learning algorithms, and they considerably influence the model's performance.

Table 2 shows the hyperparameters and the value range for each hyperparameter that is required by the five models. A grid search method was conducted next on the different combinations of the hyperparameters of each algorithm in order to explore the most suitable hyperparameter combination that helps the algorithm obtain the best performance.

**Table 2:** Initial hyperparameter value ranges and the optimal hyperparameter value for each algorithm.

Model	Hyper parameter	Definition	Value ranges	Optimal value
AdaBoost	$n$	Number of estimators	50, 100, 150, 200	50
	$\sigma$	Learning rate	${10}^{-3}$, ${10}^{-2}, {10}^{-1}$	${10}^{-1}$
XGBoost	$n$	Number of estimators	50, 100, 150, 200	50
	${d}_{tree}$	Max depth of a tree	3, 6, 9, 12, 15	9
	$\upgamma$	Min loss reduction	0, 0.1, 0.2, 0.3	0
	subsample	Subsample ratio of the training instances	0.5, 1, 2	1
LightGBM	num_leaves	Max number of nodes per tree	21, 31, 41, 51	31
	$\sigma$	Learning rate	${10}^{-3}$, ${10}^{-2}, {10}^{-1}$	${10}^{-1}$
	$n$	Number of estimators	50, 100, 150, 200	100
	${d}_{tree}$	Max depth of a tree	2, 3, 4, 5, 6	4
SVR	$\mathrm{C}$	Regularization parameter	${10}^{0}, {10}^{1}, {10}^{2}, {10}^{3}$	${10}^{0}$
	$\upgamma$	Kernel coefficient	${10}^{-6}, {10}^{-3}$,${10}^{-1}$	${10}^{-3}$
MLP	$\sigma$	Learning rate	${10}^{-3}$, ${10}^{-2}, {10}^{-1}$	${10}^{-2}$
	${nh}_{i}$	Number of neurons in hidden layer ith	50, 100, 150, 200	150
	${\mathrm{\varphi }}$	Activation function	ReLU, tanh	ReLU
	B	Batch size	8, 16, 32, 64	32

### Heat usage prediction analysis

**Figure 5:**
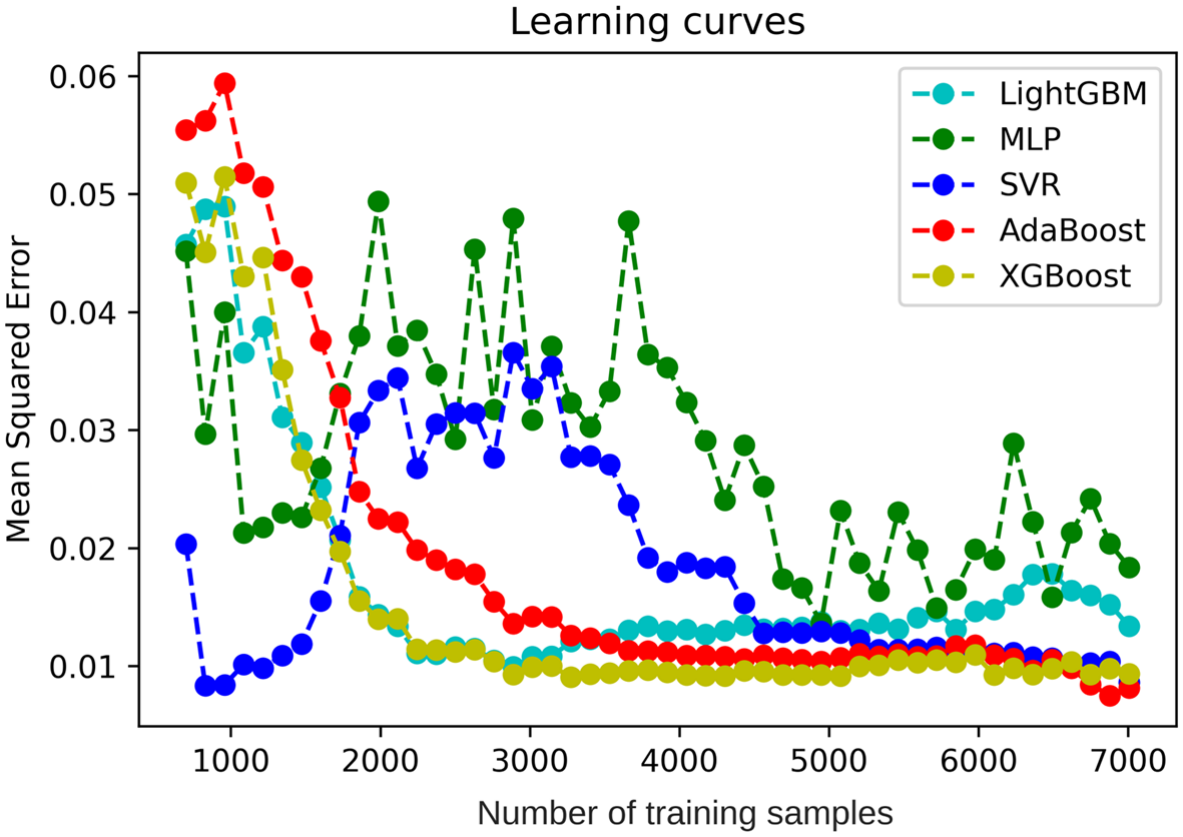
Heat demand forecasting performance using five different algorithms.

Figure 5 depicts the performance and scalability comparison of five different learning algorithms using the learning curves in order to show the effect of adding more samples during the training process. The experiment involved randomly selecting samples from the training dataset. A training sample include date, outdoor temperature, windspeed, humidity, holiday, and hourly heat demand as the features.

It can generally be concluded that SVR and MLP were highly sensitive to the dataset size, because they widely fluctuated as more training samples were added. On the other hand, the boosting algorithms, which included AdaBoost, LightGBM, and XGBoost, showed their advantages and effectiveness with a bigger dataset. The three ensemble algorithms exhibited similar trends in variation; the error gradually decreased and eventually stabilized. Low MSE scores of less than 0.02 were obtained for the three boosting algorithms when the training dataset size was over 2000 samples. XGBoost achieved the lowest mean squared error of less than 0.01 among the three algorithms, and it showed its robustness when the number of training samples reached 7000. As a result, XGBoost was utilized as the primary model for the following experiments.

Table 3 shows the heat demand forecasting performance using five ML algorithms on the test dataset. All the models generally obtained good performances on the dataset. The boosting algorithms performed better than SVR and MLP. The XGBoost algorithm achieved the highest ${\mathrm{R}}^{2}$, MSE, and MAE at 0.95, 0.12, and 0.15, respectively. On the other hand, MLP showed the lowest heat usage prediction performance with an MSE value of 0.25 and R2 at 0.89.

**Table 3:** Hourly heat load prediction performance for the five ML algorithms on the testing dataset.

Model	MAE	MSE	${\mathrm{R}}^{2}$
AdaBoost	0.16	0.14	0.94
XGBoost	0.15	0.12	0.95
LightGBM	0.18	0.17	0.91
SVR	0.24	0.21	0.92
MLP	0.23	0.25	0.89

**Figure 6:**
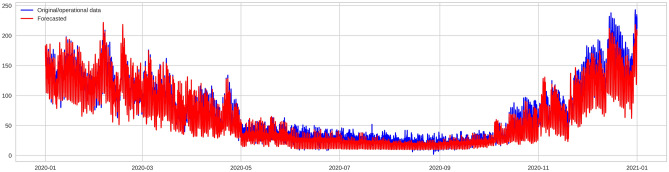
Daily heat load prediction results on the testing dataset.

Figure 6 compares the actual and the predicted heat demand for 2021 using the XGBoost model. The heat usage values predicted by the model, which are illustrated by the red line, are roughly similar to the actual heat usage values, which are illustrated by the blue line. Moreover, each month's peak and bottom heat usage were accurately predicted. However, the model performance was significantly affected, which is due to some uncommon end-user's heat usage behaviors.

### Explainable heat usage prediction

The previous section discussed what model achieved the highest heat usage forecasting performance. However, it is challenging to reveal what features are influential and how they affect the model predictions. As a result, some interesting XAI approaches are implemented in this section in order to attempt to explain how ML models predict the outcomes.

**Figure 7:**
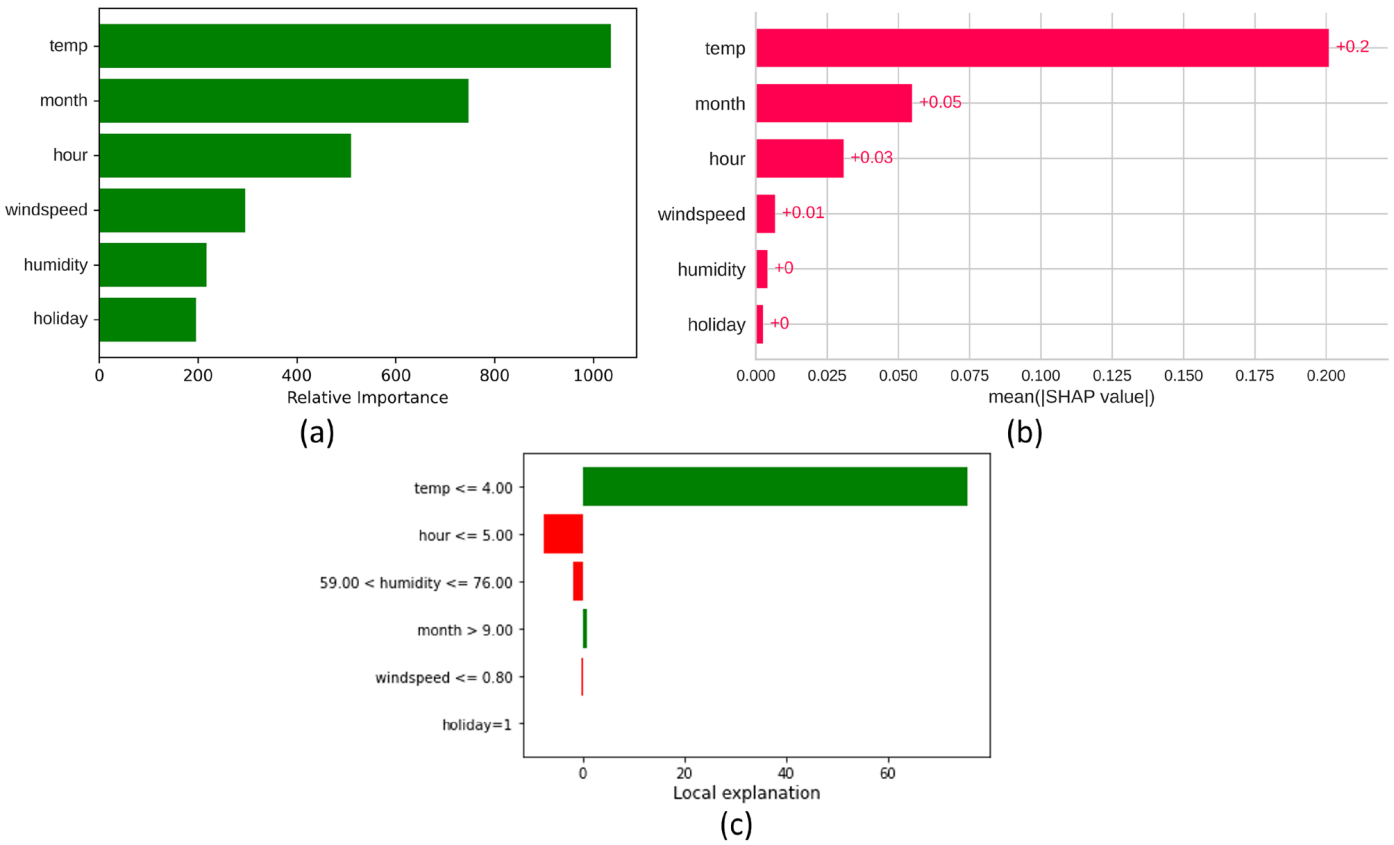
Feature importance analysis for the heat usage prediction model.

Firstly, three different feature ranking techniques were implemented in order to evaluate each feature's importance in regards to predicting the output heat usage by the model, as displayed in Fig. 7. Figure 7a calculates a feature's relative importance by examining the mean and standard deviation of impurity reduction across each tree. Figure 7b ranks the feature importance by computing the game's theoretically optimal shapley values^33^. The resulting shapley values provide a measure of the relative importance of each feature in the model prediction for a particular data point. It requires examining every possible feature combination and assessing the marginal impact of each feature on the prediction. Features with higher Shapley values are regarded as more significant. Ranking both approaches reveal that the temperature and month features are crucial, which is valid due to the end-users heat demand pattern being significantly affected by these two features.

Finally, Fig. 7c visualizes the feature importance assessed by LIME. Positive weights indicate that a feature promotes a positive prediction, while negative weights indicate the opposite. The magnitude of the weight represents the importance of the feature. It is noticeable that a temperature of 4 °C or lower (cold season) presses the model to output a higher heat usage.

The previous experiment indicated that the temperature and month features greatly impacted the model's predictions, but it did not explain exactly how the model was affected. As a result, PDP, was implemented in order to demonstrate a feature's marginal effect on the models' prediction.

**Figure 8:**
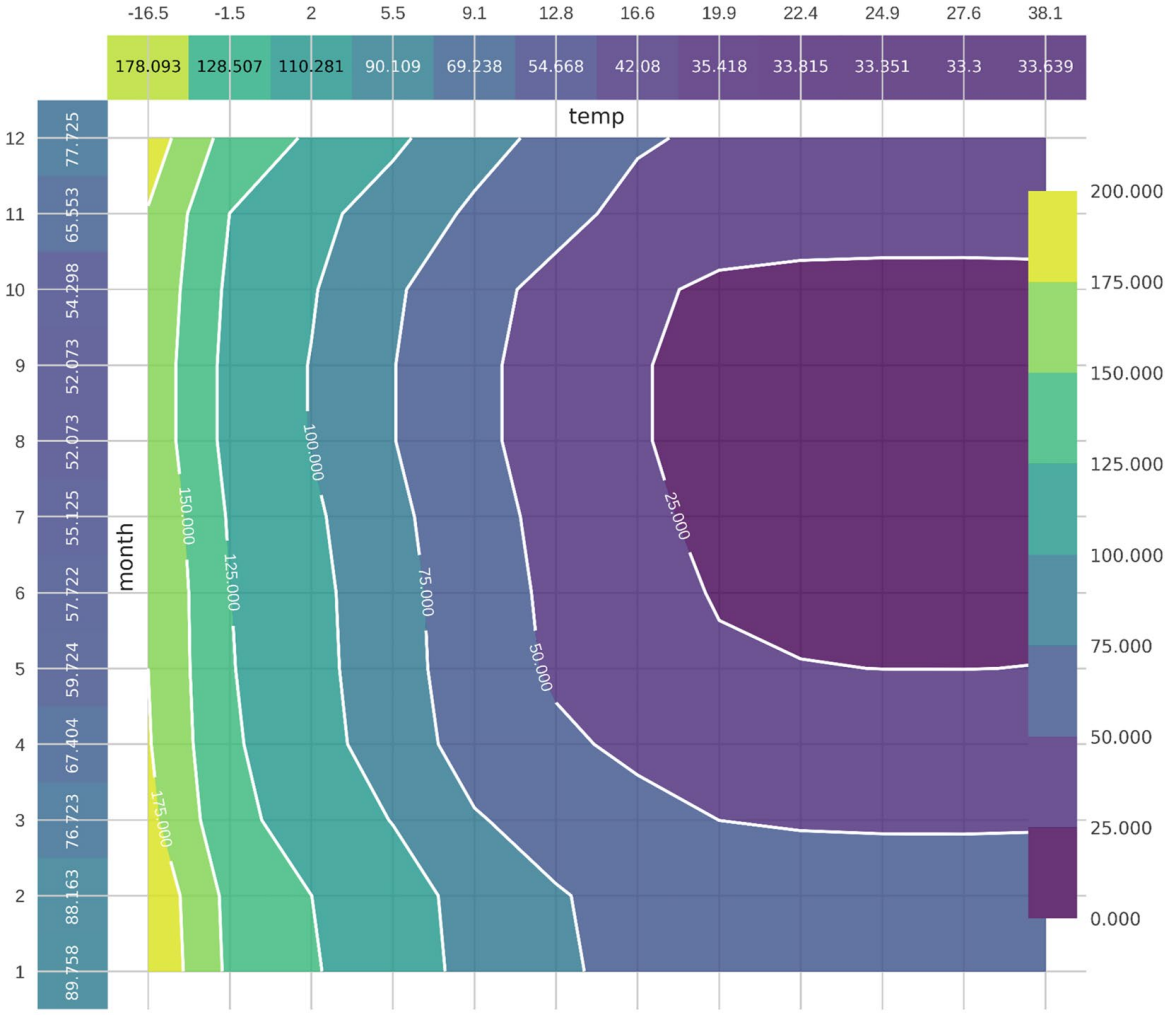
PDP interaction plot for the temperature and month features.

Figure 8 shows how temperature and month together impact heat usage in the form of contour lines. Contour was proved to work best for analyzing the impact of continuous features in the PDP interaction plot^35^.

The contour lines, ranging from 0.000 to 150.000, indicate how specific ranges of the two features affect heat usage. A higher value of the contour line implies a greater impact of the two features on heat usage. For example, during the summer season when the average temperature is above 22 °C, the features have a negative influence on the model prediction, resulting in an average heat demand of less than 50 Gcal and a contour line value of under 25.000. On the other hand, contour line values greater than 125.000, corresponding to the winter season with an average temperature of fewer than 2 °C, positively impact the model prediction leading to the average heat usage of over 120 Gcal.

**Figure 9:**
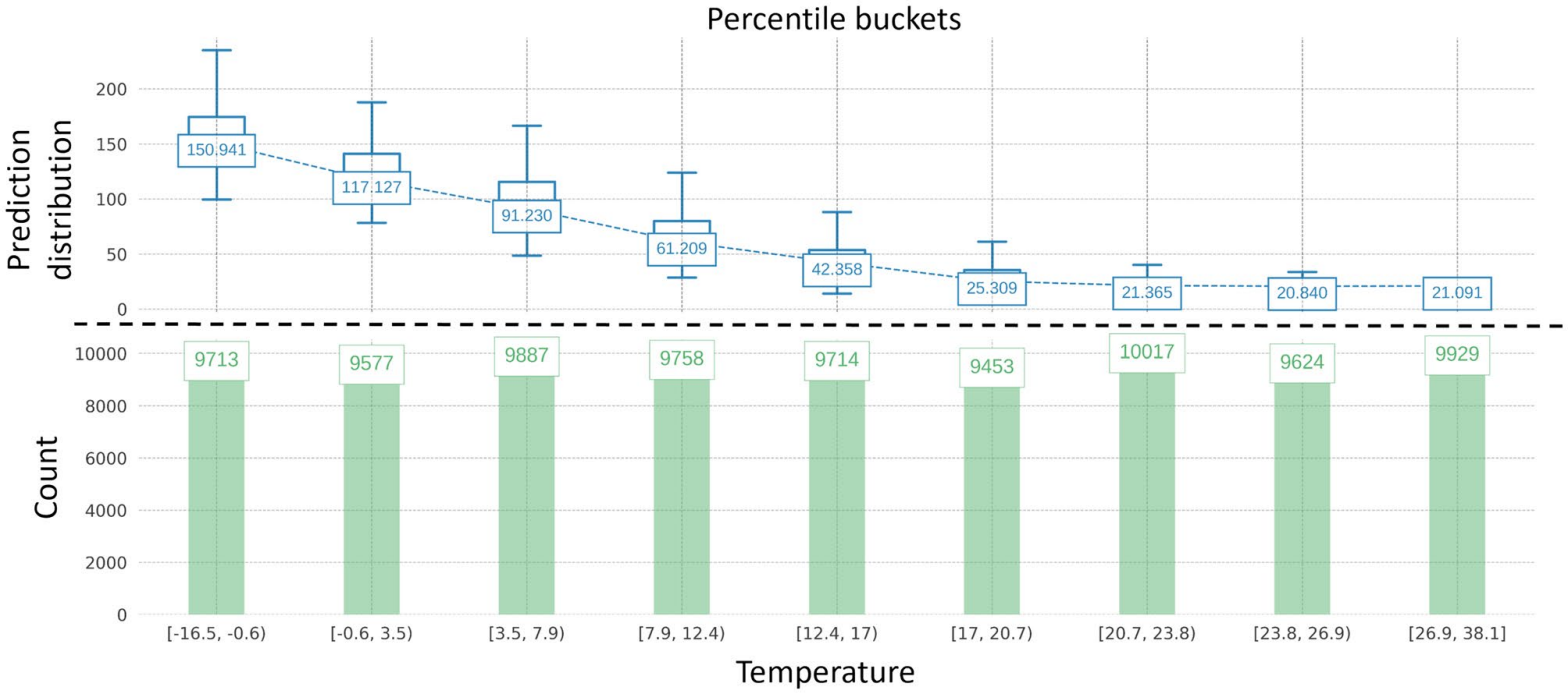
Actual predictions plot for the temperature variable. Distribution of the actual prediction via different variable values.

Figure 9 illustrates how the temperature feature affected the heat demand through the distribution of the actual heat demand via fixed values of the temperature variable. It was observable that the hourly heat load achieved the biggest average value, which was approximately 150 Gcal, occurred when the temperature feature was between -16.5 °C to -0.6 °C, indicating the winter season. Moreover, the hourly heat demand gradually dropped when the temperature rose. The lowest hourly heat demand, around 21 Gcal, was recorded when the temperature ranged from 26.9 to 38.1 °C , which corresponds to the summer season.

Based on the data, we can conclude that the hourly heat demand is directly proportional to the temperature. In the summer, DHW accounts for the majority of the heat demand. In contrast, both DHW and SH contribute to the heat demand during the winter. Additionally, the hourly heat demand is higher during the winter, with temperatures below 10 °C, and lower during the summer, with temperatures above 26 °C.

### Comparison with similar studies

Numerous studies have been conducted in the past to predict and analyze DH head demand. However, direct comparisons with these studies are difficult due to differences in DH network designs, input data, and architecture implementations or experimental setups. We use operational data from DHS to predict heat usage patterns and compare our results using the XGBoost model, which exhibits the best prediction performance. The recorded MAE value from this study was 15%, which is smaller than the reported MAE of 18.07% by Huang et al^36^. In addition, the computed evaluation metrics are also superior to the following reseach^37,38^. Specifically, the proposed XGBoost model outperforms the study suggested by Ivanko et al^38^ in terms of MSE and correlation coefficient, achieving 12% and 0.95 on the testing set, respectively, compared to MSE of 45.04% and a coefficient of determination of 0.81. In terms of the correlation coefficient, the XGBoost method also shows better hourly prediction performance than the ANN model proposed by Bünning et al^37^, with a correlation coefficient of 0.95 for one hour compared to 0.88.

## Discussion

This section provides a discussion based on our approach and the obtained results. Furthermore, a discussion about the interpretability of the study is also presented.

### Model performance

To establish the best heat demand prediction model, five different models were evaluated with varying sizes of training datasets. Then, three evaluation metrics (MSE, MAE, and ${\mathrm{R}}^{2}$) were calculated. Figure 5 demonstrates the learning trend of these models as the number of training samples increases. When the training dataset size is less than 2000, MLP and SVR exhibit the highest accuracy. However, these models have drawbacks such as the need for sequential data and extended training times, making them more suitable for applications that can handle longer training periods. On the other hand, for larger training datasets (over 2000 samples), the accuracy of the three boosting algorithms is higher. Boosting algorithms, such as AdaBoost and XGBoost, are more appropriate for granular control and frequent updating due to their short training time, stability, and forecasting accuracy. Nonetheless, all models can generate predictions swiftly (within a second) after being trained. Hence, the time required for training and retraining the models is the primary constraint for their overall implementation.

Collinearity, which refers to the correlation between predictor variables, always exists in real-world data^29^. However, the impact of collinearity on prediction models varies due to differences in principles. Previously, several approaches have been introduced to address collinearity problems, such as pre-selection based on thresholds, clustering predictors, and regularization techniques. Regularization is a method used to reduce the complexity of the SVM model and prevent overfitting^14^. Similarly, boosting-based models like AdaBoost, XGBoost, and LightGBM can effectively handle multicollinearity problems by adjusting the number of variables sampled at each split^28^, which acts as a regularization parameter. In contrast, MLP's ability to withstand collinearity is relatively weak, which may explain its relatively low accuracy.

The way in which heat is distributed varies greatly depending on the size of the DH network, and the proposed framework is appropriate for smaller networks where the behavior of customers has an impact on the load pattern. It is possible to apply the framework to other small-scale DH networks, in order to anticipate the hourly heat demand, as long as records of the hourly heat demand and environmental factors such as wind speed, humidity and temperature are available.

### Interpretability

Model interpretability for AI models refers to the ability to transform the training and testing processes into logical rules. The model's ability to display the significance and ranking of input variables^39^ allows it to exhibit interpretability. The interpretability of a predictive model is crucial in evaluating the rationality of heat demands in a DH network. A lack of conformity to accepted principles in variable importance can indicate model instability or system malfunction^4^. Boosting-based methods are highly interpretable as they do not require the interpretation of tree structures by ML professionals, and each decision corresponds to a logical rule^14^. These models can output visual results of variable importance, with the weight and rank of variables differing depending on the model's inherent principles, as displayed in Fig. 7. However, temperature and month were consistently the most influential variables, with humidity and holiday having a negligible impact, indicating the limited influence of these variables on heat usage.

On the other hand, SVR and MLP were less interpretable, with MLP being considered a black box method due to its difficulty in identifying the features extracted from each layer of the network. The use of a linear kernel function in SVR leads to a more interpretable model, but models with other kernels can be challenging to interpret^39^.

## Conclusion

Hourly heat demand forecasting is essential for heating providers to optimize heat production and heat supply operations. This research presents an hourly heat usage prediction system that is based on standard regression algorithms, and it systematically investigates the input features' influence on the models' outcomes.

First, additional weather information, which includes the outdoor temperature, wind flow speed, and humidity of the corresponding hourly historical heat demand, were extracted during the data collection process, and they were used as the input features. After that, various data preprocessing procedures were implemented in order to clean the dataset. The preprocessed dataset was utilized in order to thoroughly analyze the common heat demand patterns. Finally, the dataset was inputted into five well-known regression algorithms, namely SVR, MLP, XGBoost, AdaBoost, and LightGBM, in order to determine what model is the most suitable for the heat usage prediction task based on standard evaluation metrics.

The XGBoost model achieved the lowest MSE via various experiments, which was less than 0.01, and it was robust when the number of samples in the training dataset increased. Finally, various XAI methods, such as SHAP and PDP were applied in order to thoroughly analyze how the model gave a particular prediction. The results showed that temperature and time-related variables are the most critical features that contribute to the model's predictions.

More attention will be directed in the future toward novel heat load prediction techniques, such as multi-step ahead prediction. In addition, collecting a larger dataset with additional variables can improve the performance and efficiency of the model.

## Data Availability

The datasets used and/or analysed during the current study available from the corresponding author on reasonable request.

## Code availability

The source code for all the analyses presented in this study can be found on these GitHub repositories: https://github.com/minhdl93/HeatLoadAnalysis.
